# Evaluation of two enzyme-linked immunosorbent assays for serodiagnosis of Aleutian mink disease virus infection in mink

**DOI:** 10.1186/1751-0147-55-86

**Published:** 2013-11-25

**Authors:** Anna-Maria Andersson, Per Wallgren

**Affiliations:** 1 Department of Animal Health and Antimicrobial Strategies, The National Veterinary Institute, Uppsala, SVA SE-751 89, Sweden

**Keywords:** Aleutian mink disease virus, ELISA, VP2, Counterimmunoelectrophoresis, Mink

## Abstract

**Background:**

Aleutian disease in mink is caused by infection with Aleutian mink disease virus (AMDV). In Sweden, the infection most commonly causes classical Aleutian disease in which the immune system fails to neutralize the virus and the infection becomes persistent. Diagnosis of AMDV infection is based on serological methods that detect virus-specific antibodies. Traditionally counterimmunoelectrophoresis (CIEP) has been the preferred method, but in order to enable automation interest has been paid to other antibody detecting systems. Recently, at least two different ELISA systems that detect antibodies to AMDV have been manufactured; one is based on an *in vitro* grown AMDV as antigen, and the other system is based on the AMDV capsid protein VP2 as antigen. The aim of this study was to evaluate the two ELISA systems for detection of antibodies to AMDV using CIEP as the gold standard.

**Results:**

When employing the mean optical density of the samples from CIEP negative mink plus three standard deviations as cut-off value, the ELISA with the VP2 antigen had a sensitivity of 99.7% and a specificity of 98.3% compared to CIEP (n = 364). Analysis of samples with the AMDV-G antigen based ELISA employing an assay cut-off value based on the negative control samples, as suggested by the manufacturer, resulted in a sensitivity of 54.3% and a specificity of 93.2% with reference to CIEP as the gold standard (n = 359). When employing the mean optical density of the samples from CIEP negative mink plus three standard deviations as cut-off value, the AMDV-G ELISA had a sensitivity of 37.6% and a specificity of 98.3%.

**Conclusions:**

The ELISA system based on VP2 antigen had high sensitivity and specificity, and was concluded to be an alternative to the CIEP as a diagnostic tool for AMDV antibodies. In contrast, the AMDV-G ELISA suffered from low sensitivity when compared to CIEP.

## Background

Aleutian disease in mink is caused by infection with Aleutian mink disease virus (AMDV) [1-3]. Most commonly, the infection causes classical Aleutian disease in which the immune system fails to neutralize the virus and the infection becomes persistent [2]. Depending on the color type of the mink as well as the virus strain, the animals may show progressive weight loss, reduced reproduction, polydipsia, polyuria, anemia, melaena, neurological symptoms and death [4,5]. The disease is characterized by proliferation of plasma cells (plasmacytosis), hypergammaglobulinemia, and immune complex formations [6,7]. Anti-AMDV antibodies can be detected by counterimmunoelectrophoresis (CIEP) in the blood as early as 7 days after experimental infection [8].

Depending on the color type of the mink as well as the virus strain, infection with AMDV may also induce a persistent but non-progressive infection [1,9], or a non-persistent infection where the virus cannot be detected in the blood of the mink [10]. In both these cases, AMDV fail to cause tissue lesions. Another rare type of Aleutian disease can be seen in mink kits born from seronegative dams, where infection leads to an acute intestinal pneumonia with respiratory distress upon infection of the type II alveolar cells and a concomitant surfactant deficiency [11-13].

Diagnosis of AMDV infection is based on serological methods that detect virus-specific antibodies [8,14,15]. CIEP based on *in vitro* grown AMDV antigen (AMDV-G) has been found effective for detecting serum antibodies to AMDV [8,16], and has been referred to as a gold standard [16]. However, the method in itself is labor consuming, and interest has therefore been paid to other antibody detecting systems in order to enable automation. Recently, at least two different ELISA systems have been manufactured for the use in diagnosing Aleutian disease in mink (see Methods). The aim of this study was to evaluate the two different ELISA systems for detection of antibodies to AMDV in using CIEP as the gold standard.

## Methods

### Blood samples

Blood samples were collected from 350 mink (*Neovison vison*) of various ages and color types (non-Aleutian) originating from four AMDV infected mink farms in Sweden. Twenty five minks from an AMDV free farm were sampled to represent a population of non-infected animals. The AMDV free farm was selected on the basis of prior negative testing with either CIEP or ELISA of the breeding animals once a year for more than 30 years.

Blood was obtained by toe-nail clips and collected into glass capillary tubes; two tubes with Na-heparin and one tube without additives per animal. The capillary tubes were centrifuged at 850 gs and stored in -20°C until analysis. Due to breakage of some tubes during centrifugation, there were less than three tubes available for some animals. Consequently, all three analyses were not performed for all animals.

### Counterimmunoelectrophoresis (CIEP)

CIEP was performed with a commercial antigen (Antigen Laboratory, Danish Fur Breeders’ association, Glostrup, Denmark) with a modified protocol of a previously described method [8]. Briefly, 100 × 100 mm glass slides were coated with 17 ml of 0.8% agarose (Lonza, Basel, Switzerland) in barbital buffer, pH 8.6-9.0. The centrifuged capillary tubes were cut at the interface between the plasma and the erythrocytes. The blood plasma samples were placed undiluted, according to the instructions from the antigen manufacturer, in anodal wells and antigen in cathodal wells with positive controls included on each plate. The slides were electrophoresed for 30 minutes at 4-7 volts per cm and then viewed under indirect illumination. All samples were evaluated by two observers. A positive test result was based upon visual observation of a grey- white immunoprecipitate in the agarose gel. Weak positive reactions were re-evaluated after the gel had been soaked for 60 min in 0.9% saline and dried on a glass plate. Only samples with a remaining precipitation line after soaking were recorded as positive.

### AMDV antibody detecting ELISA systems

Blood sera and plasma from the individual mink were also analyzed for presence of antibodies to AMDV with two different ELISA systems.

Method I: AMDV-G ELISA

Blood plasma samples were analyzed using an ELISA kit, based on an AMDV-G antigen that was pre-coated in micro titer plates, according to the instructions of the manufacturer (Aleutian Disease Virus (AMDV) Antibody ELISA Test, Reference ADV3005, Scintilla Development Company LLC, Bath, Pennsylvania, USA). All reagents were provided from the manufacturer except for PBS. Briefly, the thawed plasma samples were diluted 1:100 in PBS (SVA, Uppsala, Sweden) and 100 μL of each diluted sample was added to the pre-coated plates. Positive and negative controls provided with the kit were added to two wells per plate. In addition, eight blank wells where 100 μL PBS was added to each coated well were included on each 96 wells plate. After incubation for 30 minutes at room temperature (RT), each well was washed three times with wash buffer and 100 μL conjugate (protein A-HRP) was added to each well. After 30 min of incubation at RT, the plates were again washed three times with wash buffer and 100 μL substrate was added to each well. The plates were incubated 15 min at RT in the dark and the reaction was stopped with 100 μL stop reagent. The optical density was measured at 450 nm in a Sunrise ELISA micro plate reader (Tecan Nordic AB, Mölndal, Sweden) and the mean OD of the blank wells was subtracted from each result. The cut-off value was calculated with two different methods: (1) the mean OD_450_ + 10 × SD of the negative control samples as suggested by the manufacturer, and (2) the mean OD_450_ + 3 × SD of the CIEP negative samples.

Method II: VP2 ELISA

An ELISA based on a recombinant VP2 antigen was designed, employing a previously published protocol of an ELISA test on mink serum with that antigen as a base [15]. In brief, 96-well Nunc Maxisorp immunoplates (VWR International AB, Stockholm, Sweden) were coated overnight with antigen (Finnish Fur Breeder´s association, Vaasa, Finland) diluted 1:1500 in coating buffer (50 mM NaHCO3 buffer, pH 9.6, SVA, Uppsala, Sweden) and then blocked with blocking buffer (PBS containing 1% BSA, SVA). The thawed serum samples were diluted 1:200 in dilution buffer (PBS + 0.05% Tween 20 and 0.5% BSA, SVA), added to the plate and incubated for 60 min at RT. The plates were washed with PBS-T (PBS containing 0.05% Tween 20, pH 7.4, SVA) before reagent solution (peroxidase-conjugated goat anti-cat IgG, Fisher Scientific, Gothenburg, Sweden) diluted 1:40000 in PBS was added to each well and the plates were again incubated for 60 min at RT. After washing in PBS-T, substrate (3,3′,5,5′-tetramethyl benzidine, Svanova, Uppsala, Sweden) was added to each well and the plates were incubated for 15 minutes before the reaction was stopped with 0.5 M H_2_SO_4_. The optical density was measured at 450 nm in a Sunrise ELISA microplate reader. The mean OD of the two blank wells was subtracted from each result. Reference sera (negative and positive) were included on each plate. The assay cut-off value was calculated as the mean OD_450_ + 3 × SD of the CIEP negative samples.

### Ethical approval and informed consent

The study was approved by the Swedish Ethical Committee in Uppsala (Dnr C306/10) prior to initiation.

All farms participated voluntarily in the project.

### Statistical analysis

The anti-AMDV antibody activity of the samples analyzed with the two ELISA systems are shown as mean OD_450_ values ± standard deviations. STATA (http://www.stata.com) was used to perform a *t*-test for comparison of the absorbance levels for the AMDV positive and the AMDV negative herds.

Sensitivity was calculated as the number of samples positive in both the CIEP and ELISA divided by the total number of samples positive in the CIEP [17]. Specificity was calculated as the number of samples negative in both the CIEP and ELISA divided by the total number of samples negative in the CIEP [17].

## Results

### Antibody detection with CIEP

Plasma samples from AMDV infected herds were analyzed with CIEP (n = 350). Nine weak positive reactions were re-evaluated after soaking in NaCl, and six were still deemed positive. In total, 89% of the plasma samples were positive for AMDV antibodies. In the AMDV free herd (herd E), no seropositive animals were detected (Table 1).

**Table 1:** Number and percentages of CIEP positive and CIEP negative animals for each herd

		**CIEP positive**	**CIEP negative**
**Herd**	**(n)**	**(%)**	**(%)**
*AMDV positive herds*			
A	100	92	8
B	100	93	7
C	100	86	14
D	50	84	16
Total	350	89	11
*AMDV negative herd*			
E	25	0	100
Total	25	0	100

### Anti-AMDV antibody activities in sera of healthy and infected mink using AMDV-G ELISA

In total, 359 animals were analyzed with both CIEP and AMDV-G ELISA (334 animals from the AMDV positive farms and all 25 animals from the AMDV negative farm). The mean anti-AMDV antibody activity, defined as OD_450_ values, of the CIEP negative and CIEP positive mink in the AMDV-G ELISA, are presented in Table 2. The antibody activity differed significantly (p < 0.05) between the groups.

**Table 2:** The anti-AMDV antibody activity by the AMDV-G ELISA and the VP2 ELISA of the CIEP negative and CIEP positive mink

	**(n)**	**Mean OD**_**450 **_**value ± SD**	**95% confidence interval**	**Calculated cut-off value**
*AMDV-G ELISA*				
CIEP negative mink	59	0.11 ± 0.056	0.093 - 0.12	0.28
CIEP positive mink	300	0.31 ± 0.25	0.28 - 0.33	
*VP2 ELISA*				
CIEP negative mink	58	0.043 ± 0.032	0.035 – 0.051	0.14
CIEP positive mink	306	0.91 ± 0.35	0.87 - 0.95	

**Figure 1:**
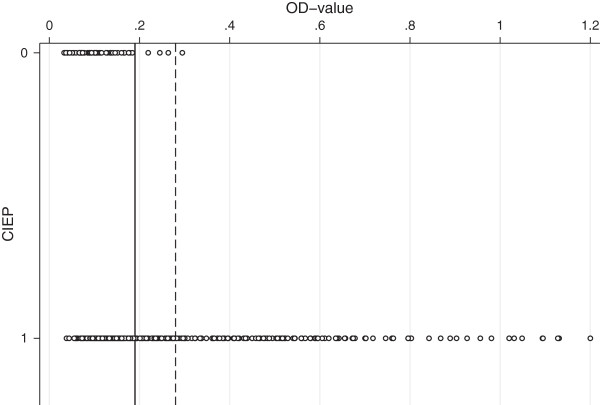
**The individual OD**_**450 **_**values obtained by the AMDV-G sorted by CIEP result.** The X axis shows the CIEP result (0 = negative and 1 = positive) and the Y axis the OD_450_ value for each of the 359 samples. The two calculated cut-off values are marked by the horizontal lines (solid line for the cut-off value suggested by the manufacturer (the mean OD_450_ + 10 × SD of the negative control samples) and the dashed line for the cut-off value based on the CIEP negative samples (the mean OD_450_ + 3 × SD of the CIEP negative samples)).

The assay cut-off value based on the negative control samples, as suggested by the manufacturer, was defined as 0.19. The AMDV-G ELISA had a sensitivity of 54.3% and a specificity of 93.2% when this cut-off value was employed (Table 3 and Figure 1). Based on this cut-off value, one of the animals from the AMDV negative farm was categorized as seropositive (OD_450_ =0.26).

**Table 3:** Comparison between detection of AMDV antibodies by ELISA and CIEP

	**CIEP positive mink**	**CIEP negative mink**	**Total**
AMDV-G-ELISA*			
Seropositive	163	4	167
Seronegative	137	55	192
	300	59	359
Sensitivity	54.3%		
Specificity		93.2%	
AMDV-G-ELISA**			
Seropositive	113	1	114
Seronegative	187	58	245
	300	59	359
Sensitivity	37.6%		
Specificity		98.3%	
VP2 ELISA**			
Seropositive	305	1	306
Seronegative	1	57	58
	306	58	364
Sensitivity	99.7%		
Specificity		98.3%	

*Cut-off value according to instruction from the manufacturer.

**Calculated cut-off value from CIEP negative mink.

The assay cut-off value based on the CIEP negative mink was defined to 0.28 (Table 2). Employing this cut-off value, the AMDV-G ELISA had a sensitivity of 37.6% and a specificity of 98.3% (Table 3). Based on this cut-off value, all of the animals from the AMDV negative farm were categorized as seronegative.

### Anti-AMDV antibody activities in sera of CIEP positive and CIEP negative mink using VP2 ELISA

In total, 364 animals were analyzed with both CIEP and VP2 ELISA (339 animals from the AMDV positive farms and all 25 animals from the AMDV negative farm). The mean anti-AMDV antibody activity, defined as OD_450_ values, of the CIEP negative and CIEP positive mink as detected by the VP2 ELISA are presented in Table 2. The antibody activity differed significantly (p < 0.05) between the groups.

**Figure 2:**
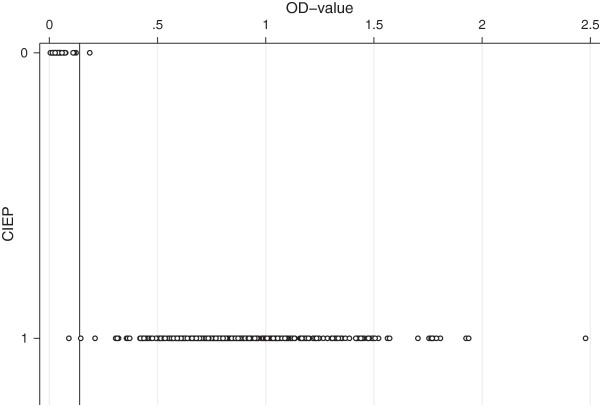
**The individual OD**_**450 **_**values obtained by the VP2 ELISA sorted by CIEP result.** The X axis shows the CIEP result (0 = negative and 1 = positive) and the Y axis the OD_450_ value for each of the 366 samples. The calculated cut-off value is marked by the horizontal line.

The assay cut-off value based on the CIEP negative mink was established to 0.14. Using this cut-off value, the VP2 ELISA had a sensitivity of 99.7% and a specificity of 98.3% (Table 3 and Figure 2). Based on this cut-off value, all of the animals from the AMDV negative farm were categorized as seronegative.

## Discussion

Serological detection of AMDV specific antibodies has been described in numerous studies. In routine diagnostics, CIEP has been the method of choice due to its low cost. With its high specificity, CIEP has also been regarded as the gold standard in AMDV diagnostics although more sensitive methods, such as radioimmunoassay and counter current line absorption immunoelectrophoresis, have been described [18,19].

Different ELISA systems have been used for diagnosing infectious diseases in many different species [20]. However, ELISA systems detecting antibodies to AMDV in mink have shown varying results. For instance, an ELISA system using fluorocarbon activated AMDV as antigen had a high sensitivity in mink with progressive AMDV, but a low sensitivity in mink with non-progressive AMDV [21]. The sensitivity of another ELISA system detecting AMDV antibodies was increased to 99% compared to CIEP in mink in Finland without compromising the specificity of the test through the use of recombinant VP2 antigen and a mid-range sample dilution [15].

In this study, we found an equally high sensitivity of the ELISA using the VP2 antigen relative to CIEP in a completely different population of mink. Only one of the samples which presented as negative in ELISA (OD_450_ = 0.09; cut-off value = 0.14) was positive with CIEP. This could reflect that the ELISA is less sensitive than the CIEP as previously suggested [21]. However, since this particular sample was one of the nine weak positive reactions in CIEP, which were re-evaluated after 30 min soaking, another possible explanation could be that it was a false positive result in CIEP and 30 min of soaking was not enough to make the weak precipitation line due to non-specific proteins disappear [19]. We also found the specificity of the ELISA using the VP2 antigen comparable to that of CIEP. Only one sample was positive in ELISA and negative with CIEP. The OD value of that sample (OD_450_ = 0.19) was only slightly above the cut-off value, indicating a low antibody titer. The sample originated from one of the AMDV positive farms and the animal was categorized as slightly seropositive in the AMDV-G ELISA as well (OD_450_ = 0.30). Since the CIEP test has been reported to have a decreased sensitivity during the early phases of infection [1,22], this sample could actually have been a truly seropositive mink and this in turn reflects the problem to find a true gold standard for AMDV serology [15]. However, this has no real implication for the test-and-remove programs in which CIEP is used at farm level today. If a farm is infected with this highly contagious parvovirus, it is unlikely that all animals will have so low antibody titers at the time of sampling that the infection in the herd will be overlooked.

When using the AMDV-G antigen and calculating the cut-off value as suggested by the manufacturer, the sensitivity (54%) and the specificity (93%) were considered as non-satisfactory for diagnostic detection of antibodies to AMDV. In order to make the evaluation of the two ELISA systems more comparable, the cut-off values for both ELISA systems were defined as the mean OD value plus three standard deviations of the CIEP negative samples. This increased the cut-off value for the AMDV-G ELISA from 0.19 to 0.28, which in turn increased the specificity from 93% to the more acceptable 98%, but decreased the sensitivity even further (from 54% to 38%; Table 3). Therefore the AMDV-G ELISA was concluded to be less applicable for the scrutinized population.

It could be questioned whether the use of either plasma or serum could have made a difference for the results. However, in the manufacturer’s instructions for both the CIEP antigen as well as the AMDV-G ELISA it is stated that the test works well both with serum and plasma even though plasma traditionally has been preferred for the CIEP. Therefore, it is unlikely that the use of plasma or serum could explain the difference between the ELISA systems. It could also be argued that some of the differences between the tests could be attributed to the difference in sample dilution. However, the sample dilution chosen was the one suggested by the manufacturer of the antigen/kit. There is always a possibility to adjust the dilution of sample, antigen and conjugate in order to achieve the optimal OD values for the micro plate reader of certain specified control substances (negative, low positive and high positive). However, at this level, we merely used previously developed systems and therefore we used the same sample dilution as suggested by the manufacturer. Further, there was no clear pattern in the results which would indicate that the inadequate sensitivity and specificity of the AMDV-G ELISA was due to differences in sample solution.

The sensitivity and specificity of diagnostic tools are important for the clinical application. However, no diagnostic test is perfect and whenever creating a cut-off value from continuous data, there will be a trade-off between sensitivity and specificity [17]. When diagnosing AMDV infection in mink, a high sensitivity of the diagnostic test is important especially when the test is used in a stamping-out program where it is essential that no infected mink escape detection, *i.e.* the sensitivity needs to be as close to 100% as possible. However, the specificity of the test must also be high. It is not desirable to have non-infected mink ending up as seropositive to AMDV since this complicates the screening of non-infected herds and makes it impossible to correctly identify newly infected farms with only a few seropositive animals. In this study, the VP2 ELISA offered both a sensitivity and specificity comparable to CIEP, indicating that the two tests detect similar virus structures, and therefore the VP2 ELISA was concluded to be the preferred ELISA method for the scrutinized mink population.

## Conclusion

This study evaluated two ELISA systems as a diagnostic tool for AMDV antibodies in mink employing CIEP as the gold standard. The ELISA system based on VP2 antigen was found to have a sensitivity and specificity comparable to CIEP, and was concluded to be a fully applicable alternative to the CIEP. In contrast, the AMDV-G ELISA suffered from low sensitivity when compared to CIEP.

## Competing interests

The authors declare that they have no competing interests.

## Authors’ contributions

AMA performed all experiments under supervisory of PW. Both AMA and PW were involved in designing the study and writing the manuscript. Both authors read and approved the final manuscript.
